# Modeling and Optimization of Directly Modulated Piezoelectric Micromachined Ultrasonic Transducers

**DOI:** 10.3390/s21010157

**Published:** 2020-12-29

**Authors:** Flavius Pop, Bernard Herrera, Cristian Cassella, Matteo Rinaldi

**Affiliations:** Electrical and Computer Engineering Department, Northeastern University, Boston, MA 02115, USA; herrerasoukup.b@northeastern.edu (B.H.); c.cassella@northeastern.edu (C.C.); m.rinaldi@northeastern.edu (M.R.)

**Keywords:** PMUT, AlN, MEMS, ultrasound, ultrasonic actuators, piezoelectric materials, direct modulation, continuous waves, implantable medical devices, implantation depth, communication range, ON/OFF keying

## Abstract

The present work details a novel approach to increase the transmitting sensitivity of piezoelectric micromachined ultrasonic transducer arrays and performing the direct modulation of digital information on the same device. The direct modulation system can reach 3× higher signal-to-noise ratio level and 3× higher communication range (from 6.2 cm boosted to 18.6 cm) when compared to more traditional continuous wave drive at the same energy consumption levels. When compared for the same transmission performance, the direct modulation consumes 80% less energy compared to the continues wave. The increased performance is achieved with a switching circuit that allows to generate a short high-AC voltage on the ultrasonic array, by using an LC tank and a bipolar junction transistor, starting with a low-DC voltage, making it CMOS-compatible. Since the modulation signal can directly be formed by the transmitted bits (on/off keying encoding) this also serve as the modulation for the data itself, hence direct modulation. The working principle of the circuit is described, optimization is performed relative to several circuital parameters and a high-performance experimental application is demonstrated.

## 1. Introduction

**Figure 1:**
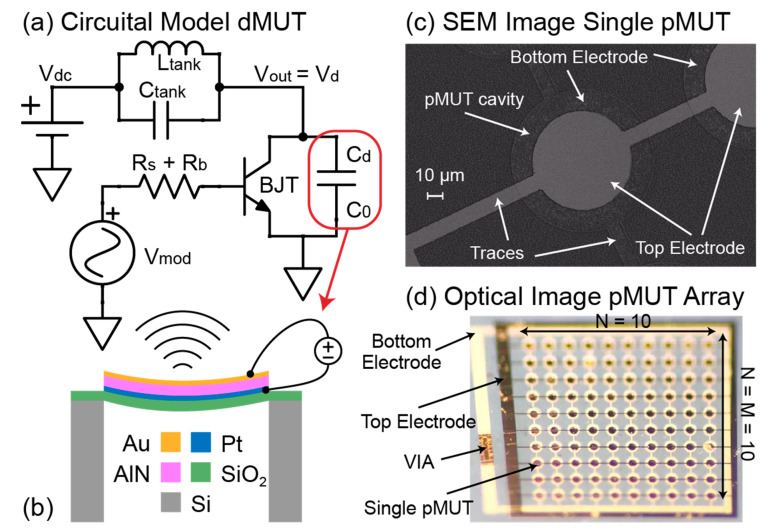
(**a**) Circuital model of the dMUT system. (**b**) Design of a single pMUT. (**c**) Scanning Electron Microscope (SEM) image of a single pMUT [19]. (**d**) Optical image of a fabricated pMUT array [19].

Ultrasonic transducers are devices that are able to generate ultrasonic waves and propagate them in either air or water-like media (water, de-ionized water, silicone oil, castor oil, tissue phantom or human tissue). In particular, Micromachined Ultrasonic Transducers (MUTs) can be of two types: capacitive MUTs or cMUTs [1,2] and piezoelectric MUTs or pMUTs [3,4]. While both devices are actuated with an electric field, the pMUTs take advantage of the piezoelectric properties as well. The generation of the ultrasonic waves is due to the displacement of a membrane that consists of a piezoelectric layer, sandwiched between two electrodes, and a structural layer, all suspended on top of a cavity trenched on the handling wafer Figure 1b. The membrane displaces due to the inverse piezoelectric effect activated by the electric field of an AC voltage signal applied between the two electrodes. The higher the voltage, the higher the membrane displacement, and thus the output pressure generated by the pMUT. These devices can be used for a wide variety of applications, such as fingerprint sensors [5,6,7], range finders [8,9,10], power transfer [11,12,13], medical imaging [14,15,16,17,18], and more recently, for intra-body and underwater communication [19,20,21,22]. In all of the above applications, pMUTs are used as transmitting and receiving elements of ultrasound pressure. In order to extend the range of transmission and minimize the power consumption, there is the need for more sensitive elements. This translates into higher output pressure given a fixed input voltage (transmitter sensitivity) and higher received voltage given a fixed input pressure (receiver sensitivity). The most commonly used technique to increase the sensitivity is to combine single pMUTs into arrays and to make the latter work as a single radiating element [23]. This allows to transmit the combined pressure and to receive the combined charge of all the individual pMUTs in the array. In this work, we propose a complementary driving circuit that can significantly increase the transmitting sensitivity of a pMUT array without any modification of the array itself.

The driving circuit proposed in this work and shown in Figure 1a is able to generate high AC voltages on top of a pMUT array starting with low voltage input signals. This allows to maintain compatibility with existing electronics, such as CMOS Integrated Circuits (IC), off-the-shelf microcontrollers, and Field Programmable Gate Arrays (FPGA). Moreover, the driving circuit allows to directly feed the information to be transmitted into the pMUT array with an ON/OFF keying modulation scheme. The data to be transmitted can be generated as a bitstream with a commercially available microcontrollers and directly fed at the input of the driving circuit, thanks to the low voltage input signal requirements. This technique is known as direct modulation and it has been demonstrated in the past for RF applications [24,25,26,27,28]. The novelty in this paper consists on applying the direct modulation to ultrasonic transducers (dMUT) and simultaneously increasing the transmission sensitivity as initially demonstrated in [29]. In this paper the following are detailed: working principle of the dMUT, gain and energy consumption optimization based on several parameters of the system and experimental results of its first implementation.

## 2. Materials and Methods

### 2.1. System Design of the dMUT

The dMUT circuit consists of an NPN Bipolar Junction Transistor (BJT), an LC filter tank and a driving capacitance, as shown in Figure 1a. The BJT is biased through a base resistance R_b_ with an AC modulation signal of amplitude V_m_ and frequency f_m_. The amplitude of the modulation signal is such that it drives the BJT in the cut-off (V_m_ = 0 V) and saturation (V_m_ = 1 V) regions, thus making it behave as a switch. The emitter of the BJT is connected to ground while its collector is connected to both the LC filter tank and the driving capacitance C_drive_. This capacitance represents a generic transducer that is modeled with a static capacitance and is voltage driven. While the dMUT can drive a generic ultrasound transducer, in this work we envision that the C_drive_ is either a single pMUT’s capacitance or the total capacitance of a pMUT array. While the C_drive_ is connected to ground, thus appearing between the collector and the emitter of the BJT, the LC tank is biased with a DC voltage source V_dc_. This source allows to keep energy stored in the tank’s filter, which consists of a capacitance C_tank_ and an inductance L_tank_. Because it is acting as a switch, the BJT turns the connection between the LC tank and the C_drive_ ON and OFF. In particular, in the ON-state (or saturation region) the BJT creates a current path between the collector and emitter, thus shorting the C_drive_ to ground, allowing it to discharge the stored energy or radiate in the case of a pMUT device. On the other hand, when going back to the OFF state (or cut-off region), the BJT has no more current flowing and the C_drive_ is suddenly connected to the LC tank, ready to re-charge again. If the tank capacitance is much smaller than the driving one (e.g., C_tank_ ≤ C_drive_/10), then we are generating an abrupt energy imbalance, that will result into the formation of a high amplitude and short duration AC pulse. This approach works because the quality factor of the pMUT array in water or tissue (Q_pMUT_ ≈ 1–5) is much smaller compared to the quality factor of the inductance of the LC tank (Q_tank_ ≈ 40–50): Q_pMUT_ << Q_tank_. This kind of AC pulse is ideal for driving ultrasonic transducers for several reasons: it increases the transmitted pressure and the operation range, it improves Signal-to-Noise Ratio (SNR), and at the same time it reduces the power consumption compared to driving the pMUT with continuous waves (CW). The generated pulse intensity can also be optimized by tuning the resonance frequency of the LC tank f_tank_ with respect to the resonance frequency of the pMUT array f_pMUT_. Finally, given the switching operation of the BJT, the input AC modulation signal can be synthesized as a square wave and encode an ON/OFF keying communication scheme, allowing the transmission of “0 s” and “1 s” directly through the dMUT system on top of an ultrasonic transducer.

### 2.2. Modeling of an Individual pMUT

**Figure 2:**
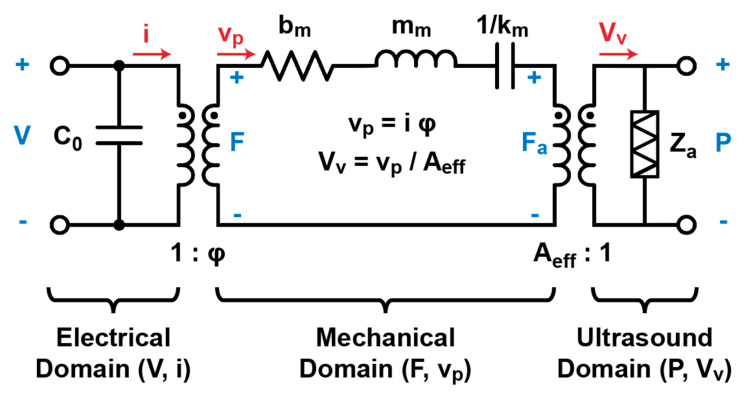
Mason circuital model of the pMUT [30,31,32]. Electrical, mechanical, and ultrasound domains are fully modeled. On one hand, the electrical domain is transformed into the mechanical domain through an ideal transformer of ratio 1:ϕ which is the electro-mechanical coupling factor. On the other hand, the mechanical domain is transformed into the ultrasound domain trough the effective area A_eff_:1 which is 1/3 of the area of the pMUT membrane.

In order to fully evaluate the performances of the dMUT system and its energy consumption, we need to simulate the full pMUT circuital model. Here we are choosing to use the Mason model [30,31,32] as shown in Figure 2 that models the electrical, mechanical, and ultrasound domain. The electrical domain contains the static capacitance C_0_ is mainly responsible for the voltage gain of the dMUT. Following we have the motional parameters such as the mechanical damping b_m_, the motional mass m_m_, and the motional stiffness k_m_, which will set the mechanical resonance frequency of the pMUT membrane and the current consumption. Finally, in the acoustic domain we have the acoustic impedance Z_a_ which is responsible for the ultrasonic radiation. The real part of this term is responsible for more current consumption. More information about the modeling of the pMUT is shown in previous publication [33].

### 2.3. Simulation of the dMUT

**Figure 3:**
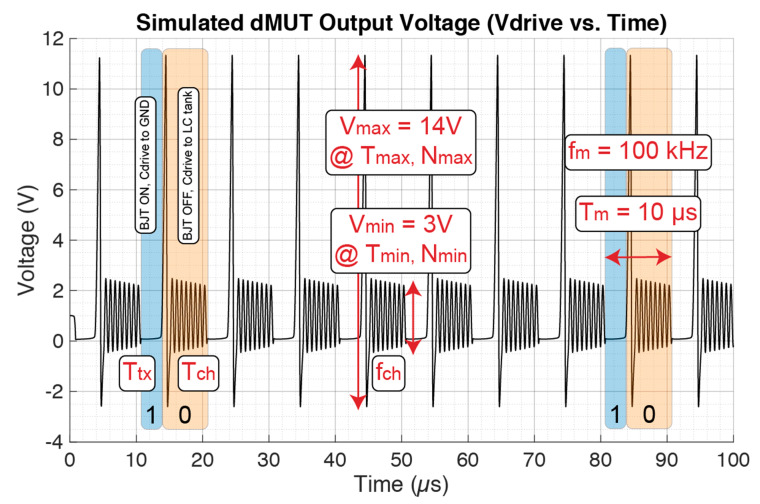
Simulation of the dMUT system with the values from Table 1. The blue region of period T_tx_ indicates the transmission region there the BJT is ON and C_drive_ is connected to ground. The orange region of period T_ch_ instead indicates the charging region where the BJT is OFF and C_drive_ is connected to the LC tank.

The full dMUT system simulation is shown in this section. The values used are presented in Table 1 together with the relationships between those parameters as discussed in the previous section. Furthermore, novel characterization metrics will be defined to evaluate the performance of a dMUT system. The simulation results of the dMUT output voltage V_drive_ over time is shown in Figure 3.

Given a modulation frequency of f_m_ = 100 kHz, the resulting modulation period will be T_m_ = 1/f_m_ = 10 μs. The T_m_ is highlighted in the figure with a “blue” or transmission region, and an “orange” or charging region. In the transmission region the BJT is ON and C_drive_ is connected to ground for a period T_tx_ ≈ 3 μs. During this time, the capacitance discharges, or in the case of a pMUT, it radiates the power outside the system, thus we have ultrasonic transmission, which can be encoded with an “1” bit. On the other hand, in the charging region the BJT is OFF and C_drive_ is connected to the LC tank for a period of T_ch_ ≈ 7 μs. During this time, energy stored in the LC filter flows into the driving capacitance or pMUT, thus charging it, which can be encoded with a “0” bit. Due to an abrupt capacitance and frequency change when the LC tank connects to the C_drive_, there is the generation of a short time and high amplitude AC pulse, which in this case is V_max_ = 14 V for a period of T_max_ ≈ 1 μs and a number of cycles N_max_ = 1. Following this short pulse, the signal’s intensity decays to a lower amplitude of V_min_ = 3 V for a period of T_min_ ≈ 6 μs and a number of cycles of N_min_ = 8. These parameters and their dependencies are going to be analyzed further in the paper.

Now we can define some time and intensity-based performance metrics for the dMUT system. First, we have the transmission Duty Cycle (DT) defined as following:

(1) $$\mathrm{DT}=\;\frac{T_{\mathrm{tx}}}{T_{m}}=1-\frac{T_{\mathrm{ch}}}{T_{m}}$$

Based on the values obtained from the simulation the DT ≈ 30%. Second, the Pulse Time Generation (PTR) ratio is defined relating the decay period to the charging period:

(2) $$\mathrm{PTR}=\frac{T_{\max}}{T_{\min}}$$

Based on the values obtained from the simulation the PTR ≈ 17%. Third, the communication bandwidth (BW) for an ON/OFF keying modulation scheme is defined as follows:

(3) $$\mathrm{BW}=N_{\mathrm{bit}}\cdot f_{m}=2\;\left[\mathrm{bits}\right]\cdot 100\;\left[\mathrm{kHz}\right]=200\;\left[\mathrm{kbits}/\sec\right]$$

At this point, the pulse amplitude gain with respect to the input modulation voltage, which is the gain of the system, can be defined for each modulation frequency f_mod_ as:

(4) $$A_{\max}=\;{\frac{V_{\max}}{V_{\mathrm{mod}}}|}_{f_{\mathrm{mod}}}$$

Based on the values obtained from the simulation the A_max_ ≈ 14 V. For the pulse amplitude gain a logarithmic value G can be defined as:

(5) $$G=20\cdot {\mathrm{Log}}_{10}\left(A_{\max}\right)$$

Based on the value of A_max_, G ≈ 23 dB. Finally, the charging operation frequency f_ch_ which is set by the combined C_drive_ and the C_tank_ can be identified as:

(6) $$f_{\mathrm{ch}}=\;\frac{1}{2\pi \cdot \sqrt{L_{\mathrm{tank}}\cdot \left(C_{\mathrm{tank}}+C_{\mathrm{drive}}\right)}}$$

Based on the values from Table 1, f_ch_ ≈ 1.5 MHz, confirmed by the simulation in Figure 3.

## 3. Results

### 3.1. Energy Consumption

**Figure 4:**
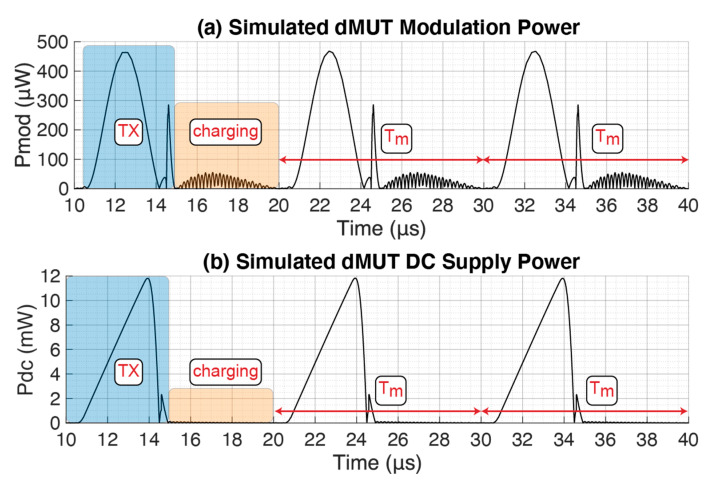
Power consumption of the dMUT system with the values from Table 1. (**a**). Power generated by the AC sinusoidal input modulation signal. (**b**). Power generated by the DC bias voltage signal.

In this section we are going to compute the energy consumption of the dMUT system and compare it with the equivalent case of driving and modulating the pMUT array with the standard continuous wave (CW) approach. As shown in Figure 1a, the dMUT has only two voltage sources, V_mod_ and V_dc_, which will set the power consumption for the circuit. In Figure 4 we are showing the dMUT power consumption when driving a 20 × 20 array. Since the waves are distorted, we are going to integrate the power over a modulation period T_m_ in order to determine the energy consumption, as following:

(7) $$E=\int_{0}^{T_{m}}P\cdot \mathrm{dt}$$

**Figure 5:**
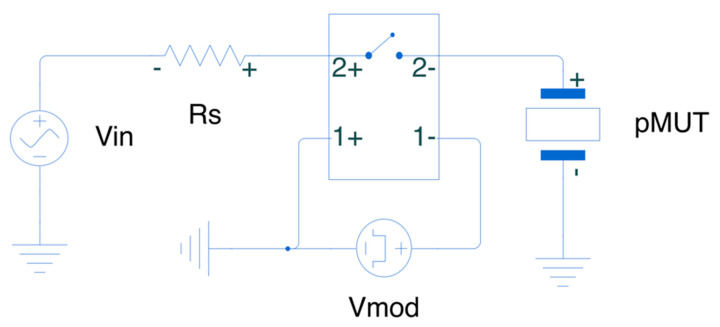
Continuous wave driving of the pMUT array with and ideal switch to implement the ON/OFF keying modulation.

The resulting modulation energy is E_mod_ ≈ 1.15 nJ and the DC supply energy is E_dc_ ≈ 24.85 nJ, for a total energy of E_tot_ ≈ 26 nJ to drive the entire array. If normalized by the number of elements, we obtain a required energy per driven pMUT of ${\frac{E_{\mathrm{tot}}}{\mathrm{pMUTs}}|}_{\mathrm{DMUT}}\approx 65\;\mathrm{pJ}$. At this point we compare the energy consumption with the CW approach shown in Figure 5.

In order to have the same performance as the dMUT, the CW circuit needs to produce the same peak-to-peak voltage on the driven pMUT. In this case V_pp_ = 14 V as shown in Figure 3, thus we are going to assign this value to V_in_ = 14 V (or 7 V AC source) in Figure 5 operating at the resonance frequency of the pMUT array, f_in_ = fpMUT. On the other hand, the voltage driven switch is ideal in the simulation, thus V_mod_ has no power consumption. However, the V_mod_ frequency of f_mod_ = 100 kHz will set the period T = 10 μs, interval for which Vin is on, thus setting its energy consumption.

By simulating the pMUT Mason model as shown in Figure 2, we obtain that the current consumption for a single pMUT is I_in_ ≈ 4 μA. For the switch instead, we can assume a low power consumption Single Pole Single Throw (SPST) such as P_mod_ ≈ 1 μW (ADI ADG902), resulting in an energy consumption E_mod_ ≈ 10 pJ. Finally, the input energy can be computed over the modulation period resulting being E_in_ ≈ 107 pJ, thus the total energy consumption per pMUT is ${\frac{E_{\mathrm{tot}}}{\mathrm{pMUTs}}|}_{\mathrm{CW}}\approx 117\;\mathrm{pJ}$. This makes the dMUT system 45% more efficient in terms of energy consumption, or the CW systems is 80% more energy demanding.

### 3.2. System Optimization

This section focuses on how the dMUT system scales when changing the driving capacitance. As this represents the equivalent static capacitance of a pMUT array, the scaling corresponds to changing the total number of individual pMUTs in the array. Furthermore, how to optimize the system performance, gain and energy consumption will be shown in terms of input modulation frequency f_m_, capacitance ratio CR, frequency ratio between pMUT frequency and LC tank filter frequency FR and DC bias of the filter V_dc_. Each of those parameters will be swept once at a time while all the other parameters in the dMUT system are kept constant as in Table 1.

#### 3.2.1. Modulation Frequency (f_m_)

**Figure 6:**
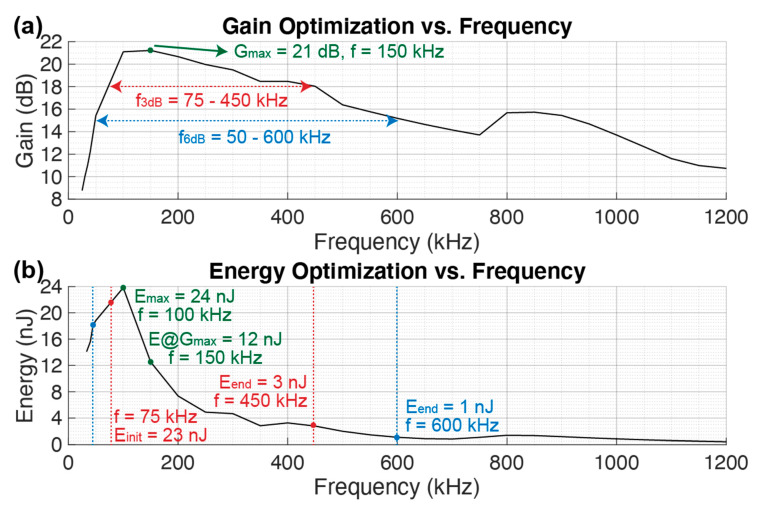
Gain (**a**) and energy (**b**) optimization over a sweep on the modulation frequency f_m_. The remaining values are maintained constant as in Table 1.

Figure 6 shows the dMUT system gain and energy consumption while sweeping the modulation frequency f_m_. This allows to choose the optimal f_m_ of operation that ultimately sets the data-rate in an ON/OFF modulation communication scheme. The maximum gain G_max_ = 21 dB is obtained at fm = 150 kHz as shown in Figure 6a. In order to optimize the energy consumption per modulation period (or every two transmitted bits), the operation of the system is extended to two regions R_3dB_ and R_6dB_, corresponding to 3 dB and 6 dB distance from G_max_ respectively. From Figure 6b it can be seen that the energy consumption reaches a maximum E_max_ = 24 nJ at f_m_ = 100 kHz and then it decreases with an inverse quadratic law while increasing linearly the f_m_. By operating at the far right of the two operation regions defined above, the energy is reduced to E_3dB_ = 3 nJ and E_6dB_ = 1 nJ at f_m_ = 450 kHz and f_m_ = 600 kHz.

In order to understand the physical reason behind the energy decrease with increased frequency, the power consumption curve in Figure 4 needs to be studied. First of all, since the considered energy is the energy per modulation period T_m_, by increasing f_m_ we reduce the T_m_, thus when integrating to compute the energy, this will be lower. Second of all, there will be a major integration reduction of the area in the transmission region P_tx_, since we are shorting the BJT faster, and thus it consumes less power and energy.

#### 3.2.2. Array Dimension (N × N)

**Figure 7:**
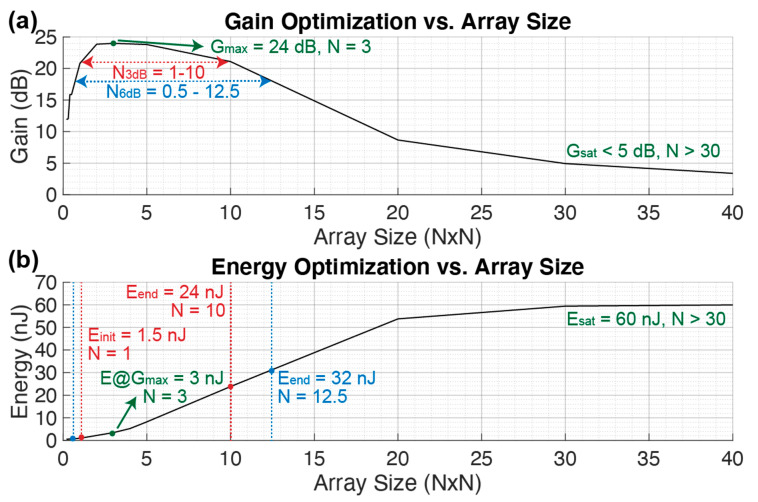
Gain (**a**) and energy (**b**) optimization over a sweep on the array size NxN. The remaining values are maintained constant as in Table 1.

Figure 7 shows the gain and energy consumption while sweeping the array size N_tot_ = N · M = N^2^ for N = M. The array size sets the total driving capacitance based on the evaluated capacitance of a single pMUT element C_d_ = N_tot_ · C_0_. On one hand, Figure 7a shows a maximum gain of G_max_ = 24 dB when the array size is N = 3. On the other hand, Figure 7b shows a linear energy consumption increase while increasing N. By extending the operation regions to R_3dB_ and R_6dB_, instead of optimizing the energy consumption, the maximum energy while operating within the same regions can be determined. In particular the maximums are E_3dB_ = 24 nJ and E_6dB_ = 32 nJ for N = 10 and N = 12.5. Finally, when N > 30, the gain reaches a saturation level G_min_ < 5 dB and a saturation of the energy consumption E_sat_ = 60 nJ.

In this case, the energy consumption intuitively increases with the number of elements to drive since the system will require more current to drive each pMUT. Furthermore, once the gain reaches a saturation level G_sat_ < 5 dB for N > 30, an energy saturation E_sat_ = 60 nJ can be noticed, which would equal the energy required to drive the Cd without the dMUT since the circuit has become inefficient.

#### 3.2.3. Capacitance Ratio (CR)

**Figure 8:**
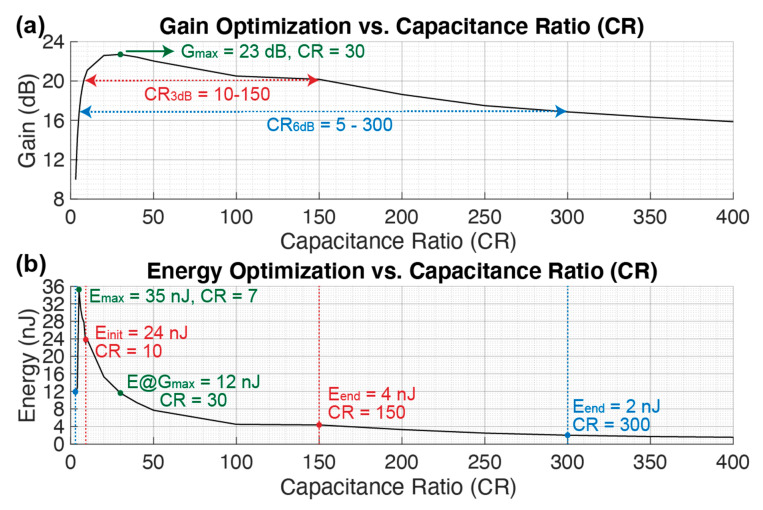
Gain (**a**) and energy (**b**) optimization over a sweep on the capacitance ratio CR. The remaining values are maintained constant as in Table 1.

In Figure 8 the gain and energy consumption are shown while sweeping the capacitance ratio CR. By increasing the CR, the dMUT gain reaches a maximum of G_max_ = 23 dB at CR = 30 and after this point the gain decreases linearly as shown in Figure 8a. Working at higher CR helps to decrease the energy consumption as shown in Figure 8b. In particular, after reaching a maximum of E_max_ = 35 nJ at CR = 7, the energy decreases with an inverse quadratic law while increasing CR linearly. At this point, by choosing a point at the end of the operation regions R_3dB_ and R_6dB_, a minimum in the energy consumption per modulation period is seen, E_3dB_ = 4 nJ and E_6dB_ = 2 nJ respectively at CR = 150 and CR = 300.

The energy consumption finds its maximum when the energy exchange between the LC filter tank and the driving capacitance is not optimal and it requires additional current feeding from the DC voltage source. Once the CR is increased, the filter capacitance C_tank_ becomes much smaller than the C_d_. This permits to charge the C_d_ with more energy efficiency, explaining the decrease of the energy consumption while increasing CR.

#### 3.2.4. Frequency Ratio (FR)

**Figure 9:**
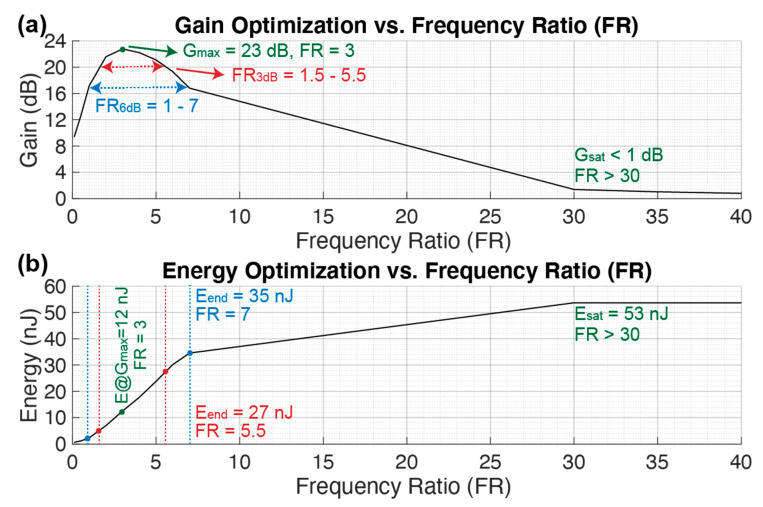
Gain (**a**) and energy (**b**) optimization over a sweep on the frequency ratio FR. The remaining values are maintained constant as in Table 1.

Figure 9 shows the gain and energy consumption while sweeping the frequency ratio FR. The gain reaches a maximum of G_max_ = 23 dB at FR = 3 and then decreases linearly until FR = 30, after which there is a gain saturation G_sat_ < 1 as shown in Figure 9a. Instead, the energy consumption increases with a square root law while increasing the FR linearly. The minimum energy consumption is obtained at the beginning of the R_3dB_ and R_6dB_ resulting in E_3dB_ = 5 nJ and E_6dB_ = 2 nJ respectively at FR = 1.5 and FR = 1. In this last parameter sweep, the energy increases while increasing FR. This physically means that the LC tank filter is operating at higher frequency with respect to the resonance frequency of the pMUT array f_pMUT_, thus it requires more energy for the filter to operate and charge up. Finally, the energy reaches a saturation level E_sat_ = 53 nJ when FR > 30, similar to the N sweep in Figure 7. This happens because the gain also reaches a saturation G_sat_ < 1, making the dMUT system very inefficient, thus the resulting energy consumption is the one that is required to drive the C_d_ without the dMUT circuit.

A summary of the values obtained from the optimization simulations are shown in Table 2.

#### 3.2.5. High Performance dMUT

**Figure 10:**
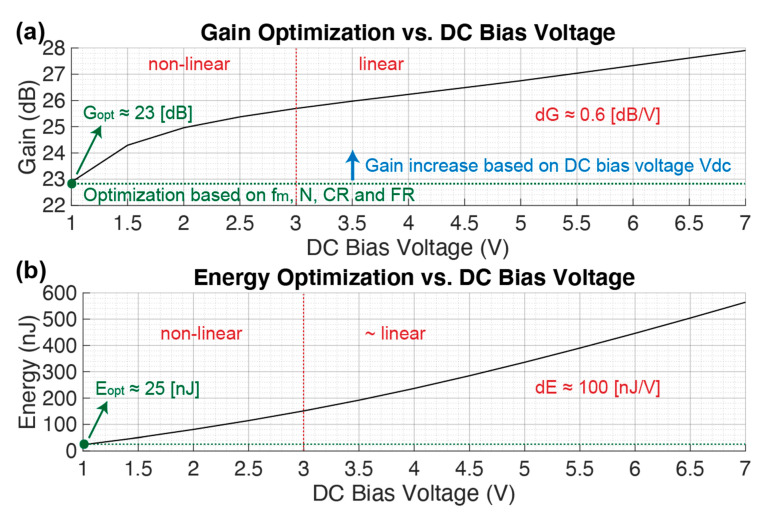
High performance gain (**a**) and energy (**b**) consumption based on the DC biasing voltage Vdc of the LC tank filter. The remaining values are maintained constant as in Table 1.

The previous sections detailed a gain and energy consumption optimization of the dMUT system by exploring the operation ranges for several parameters such as f_m_, N, CR, and FR while maintaining the remaining parameters constant as in Table 1. In particular, the DC biasing voltage of the LC filter tank was kept to V_dc_ = 1 V. In this section, a high-performance system is explored which is boosted by an increase in the DC bias. By doing so, the LC tank will be able to store more energy and boost the output voltage on top of the driven capacitance, which is the equivalent capacitance of a pMUT array. In Figure 10 the dMUT gain G for a DC bias range of V_dc_ = 1–10 V is shown. As it can be noticed, for V_dc_ = 1 V there is a minimum optimal gain corresponding to the one obtained in the optimization section. By further increasing the bias, the gain increases non-linearly up to V_dc_ = 3 V and then follows a linear trend. For the linear region we can compute the differential gain increase based on V_dc_ as following:

(8) $$\mathrm{dG}=\frac{\Delta G}{\Delta V_{\mathrm{dc}}}\approx 0.6\;\left[\frac{\mathrm{dB}}{V}\right],\;V_{\mathrm{dc}}>3\;V$$

The gain boosting comes at a large expense in the energy consumption as shown in Figure 10b. As for the gain, the energy has a minimum that comes from the optimization section E_opt_ = 25 nJ at V_dc_ = 1 V. The energy consumption follows a non-linear trend up to V_dc_ < 3 V and then a linear trend. For the linear region we can compute the differential energy increase based on V_dc_ as following:

(9) $$\mathrm{dE}=\;\frac{\Delta E}{\Delta V_{\mathrm{dc}}}\approx 100\;\left[\frac{\mathrm{nJ}}{V}\right],\;V_{\mathrm{dc}}>3\;V$$

Even though the high performance dMUT system is power hungry, it could still be an optimal solution for certain application that allows for large batteries or for wired connections. For example, this could be the case of a power transfer application from an external ultrasonic transducer, which can be wire-connected to drain power, that sends power to a medical device deeply implanted in a human body (>10 cm).

## 4. Discussion

**Figure 11:**
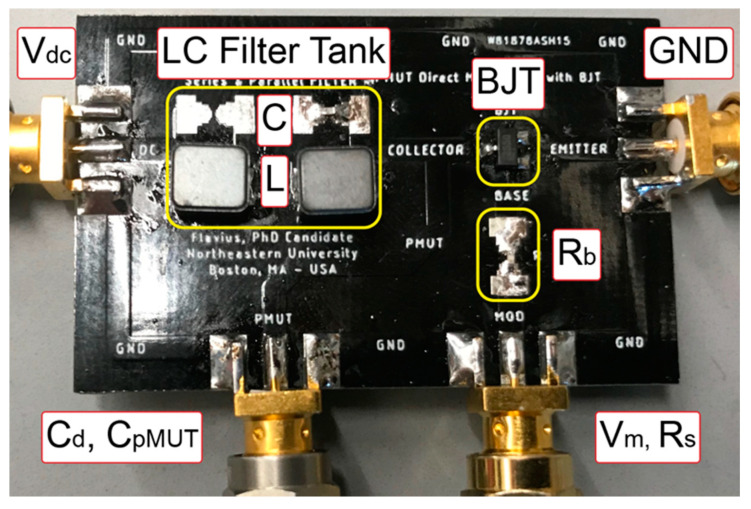
Printed Circuit Board (PCB) implementation of the dMUT system based on the parameters in Table 1 with the exception of the DC biasing voltage that was increased to V_dc_ = 7 V for high performance application [29].

In this last section the dMUT system is implemented in a Printed Circuit Board (PCB) with off-the-shelf components as shown in Figure 11. On the PCB we can notice the LC filter tank, with a capacitance C_tank_ and inductance L_tank_, the BJT and the base resistance R_b_. The chosen BJT is the ComChip MMBTA44-G since it can handle high voltages (>100 V) compared to other transistors. Moreover, the conduction losses can be estimated from the saturation voltage, V_CEsat_ ≈ 55 mV. Base on the current consumption that can range from 1 to 10 mA, the power will be around 55 to 550 μW. Following, the PCB connects through SMA connectors to the DC bias source V_dc_, to the pMUT array static capacitance C_pMUT_ or an equivalent driven capacitance C_d_, to the input modulation signal V_m_ that has a series input resistance R_s_ and to an SMA connecting the BJT’s emitter to ground GND. While this approach can work with a MOSFET, we choose a BJT because it can handle higher voltages on the collector node.

**Figure 12:**
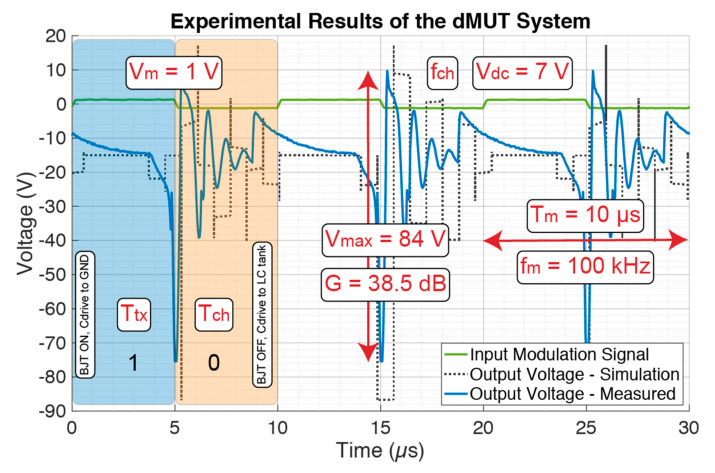
Experimental results of the dMUT system based on the parameters in Table 1 with the exception of the DC biasing voltage that was increased to V_dc_ = 7 V for high performance application. The output voltage is compared to the simulation results showing a good matching. The maximum voltage is V_max_ = 84 V which corresponds to a system gain of G = 38.5 dB of the dMUT system.

In Figure 12 the experimental results of the dMUT system are shown based on the values from Table 1 with the exception of the DC biasing voltage that is increased to V_dc_ = 7 V for high performance applications. The “green” curve shows the input modulation signal V_m_ = 1 V at f_m_ = 100 kHz. Further, the “black-dotted” curve shows the simulation of the output voltage generated on top of the driven capacitance of the dMUT array. This curve is a good match with the experimentally measured output voltage indicated by the “blue” curve. The output voltage shows a maximum of V_max_ = 84 V when a DC bias of V_dc_ = 7 V is applied to the LC filter corresponding to a system gain of G = 38.5 dB.

**Figure 13:**
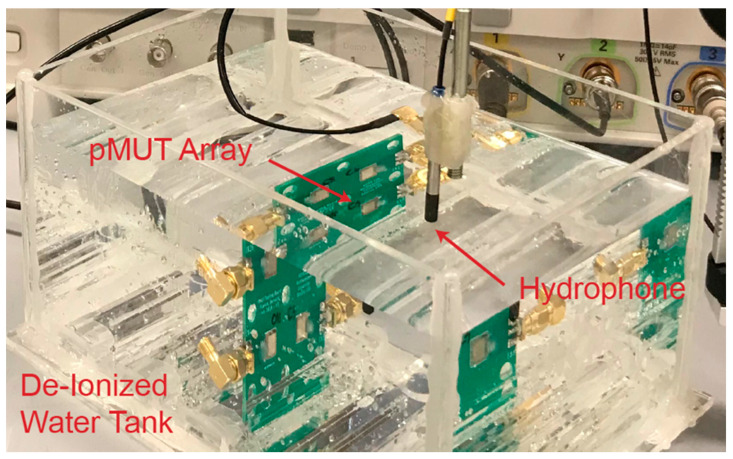
De-Ionized (DI) water tank for ultrasonic experiment measurements. The transmission is done between a pMUT array and an ultrasonic hydrophone (Teledyne TC4038) for reference. Given the small dimensions of the tank, there will be some interference due to the reflections at the interface, creating a multipath. Absorptions layers can be added to the wall to reduce the multipath phenomena.

In order to demonstrate the advantage of driving a pMUT array with the dMUT system as opposed to traditional CW, we are going to perform an ultrasonic transmission experiment in a De-Ionized (DI) water tank as shown in Figure 13. The pMUT array is used as a transmitter and is driven either by the dMUT system or by a simple CW system. The received signal is then received and captured by an underwater ultrasonic hydrophone (Teledyne TC4038) and converted into electrical signal. The sensitivity of such device is −228 dB reference to 1 V/μPa, which sets the minimum detectable signal. Translated into electrical signal, this means that the Noise Floor (NF) is NF = 4 μV peak-to-peak, thus the incoming ultrasonic signal needs to generate a signal higher than the NF in order to be detectable.

**Figure 14:**
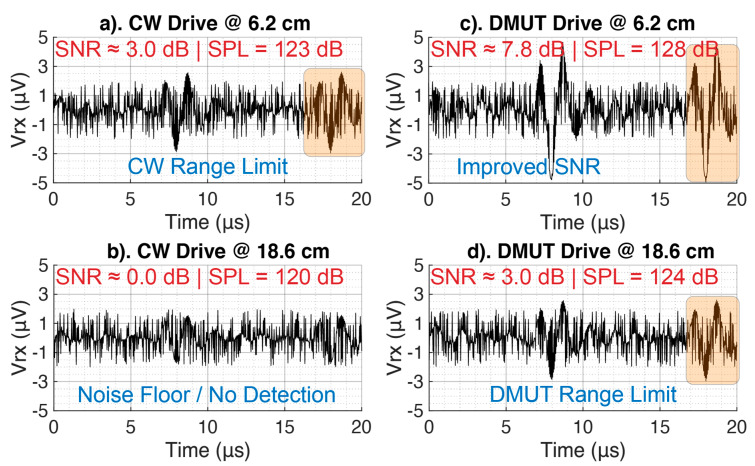
Measurement results in a water tank. Received signal captured by a Teledyne hydrophone with −228 dB re 1 V/μPa corresponding to a noise floor of NF = 4 μPa. Both systems are driven with the same energy levels. (**a**) CW drive at 6.3 cm corresponding to the maximum reachable distance where the SNR is at least 3 dB. (**b**) CW drive at 18.6 cm where the SNR is 0 dB, and the signal is not detectable from the noise. (**c**) DMUT drive at the same distance as in (**a**); this new drive approach shows an improvement of the SNR from 3 dB to 7.8 dB for same input energy level. (**d**) DMUT drive at the same distance as in (**b**); this new drive approach shows an improvement of the SNR from 0 dB to 3 dB allowing, extending the communication link from 6.2 cm, which is the maximum limit for the CW drive as shown in (**a**), to 18.6 cm, which is the maximum limit for the DMUT drive as shown in (**d**), for the same input energy level.

Firstly, we drive the pMUT array in CW with the same energy level of the dMUT system and we identify the maximum reachable D_max_ at which we can still detect the incoming ultrasonic signal beyond the NF. In particular we consider a valid signal is this is 3 dB higher than the NF in terms of Sound Pressure Level (SPL), which means that the Signal-to-Noise Ratio (SNR) is SNR ≈ 3 dB. The noise level in terms of SPL is NF ≈ 120 dB. Based on this, a valid signal needs to reach SPL ≥ NF + SNR. In Figure 14 we are sowing the limit of the CW for an array of 20 × 20 pMUT at a distance ${D_{\max}|}_{\mathrm{CW}}\approx 6.2\;\mathrm{cm}$.

Secondly, we drive the pMUT array with the dMUT system at the same energy level as the CW drive. At the same maximum distance as the CW, the dMUT system shows higher SPL ≈ 128 dB and in particular the SNR ≈ 7.8 dB as shown in Figure 14c. This corresponds to an improvement of 2.8 in dB scale or 3× in linear scale.

Finally, we want to find the range limit for the dMUT drive of the array similar to the CW drive. As shown in the measurements in Figure 14c, the new maximum reachable distance with an SNR ≈ 3 dB is ${D_{\max}|}_{\mathrm{DMUT}}\approx 18.6\;\mathrm{cm}$. This corresponds to an improvement in the operation range of 3× fold. For comparison, in Figure 14b we are driving the array with the CW at the maximum distance of the dMUT. As we can see the incoming ultrasonic signal is no longer detectable, thus SNR ≈ 0 dB.

In conclusion the dMUT system was proven to be effective in increasing the transmitting sensitivity of a pMUT array while performing direct modulation of an ON/OFF keying modulation scheme and direct screaming of the bitstream. The main operating circuit of the dMUT was described in terms of its switched LC tank working principle and gain, power and energy metrics were introduced. Optimization was then performed relative to the input modulation frequency, array dimensions (drive capacitance), drive to tank capacitance ratio, and tank to pMUT resonance frequency ratio. Taking this optimization into consideration, an experimental high-performance implementation was shown able to achieve a 38.5 dB gain (a maximum of 84 V for a 1 V input) for a 7 V DC bias and a 100 kHz modulation frequency. This system can work with DC biases below 3 V which can be CMOS compatible. Furthermore, this improved performance comes with 80% less energy consumption compared to traditional CW driving and implementation of the same modulation scheme. Finally, when compared with the same energy levels, the dMUT shows an improvement of 3× of the SNR and the maximum reachable communication range at 3 dB from the noise floor. This work will be further extended in a separate paper to incorporate a full receiver architecture to demodulate the incoming signal from the dMUT and pMUT ultrasonic link. This will make use of an envelope filter, active filtering blocks, and a comparator. In the end, the transmitter and receiver will be interfaced with a microcontroller for data encoding and decoding.

## Figures and Tables

**Table 1:** Parameters summary of the dMUT system together with their typical values (unless otherwise specified in the paper) and their description.

Parameter	Value	Description
V_m_	1 [V]	AC input voltage amplitude
f_m_	100 [kHz]	Modulation frequency
R_s_	50 [Ω]	Input source resistance
R_b_	1 [kΩ]	BJT base biasing resistance
V_dc_	1 [V]	DC biasing voltage of LC tank
f_pMUT_	1 [MHz]	Resonance frequency of pMUT
FR	$\frac{f_{\mathrm{tank}}}{f_{\mathrm{pMUT}}}=5$	LC tank to pMUT frequency ratio
f_tank_	$\mathrm{FR}\cdot f_{\mathrm{pMUT}}=5\;\left[\mathrm{MHz}\right]$	LT tank resonance frequency
N, M	N = 10, M = 10	Rows and columns of single pMUT
C_pMUT_	0.5 [pF]	Static capacitance of single pMUT
C_drive_	$N^{2}\cdot C_{\mathrm{pMUT}}=50\;\left[\mathrm{pF}\right]$	Driving capacitance of dMUT
CR	$\frac{C_{\mathrm{drive}}}{C_{\mathrm{tank}}}=10$	LC tank to driving capacitance ratio
C_tank_	$\frac{C_{\mathrm{drive}}}{\mathrm{CR}}=5\;\left[\mathrm{pF}\right]$	LC tank capacitance
L_tank_	$\frac{1}{C_{\mathrm{tank}}\cdot {\left(2\pi \cdot f_{\mathrm{tank}}\right)}^{2}}\approx 0.3\;\left[\mathrm{mH}\right]$	LC tank inductance

**Table 2:** Summary of dMUT gains and energy optimization parameters and their values.

Parameter	Sweeping Variables (SV) [U]			
	f_m_ [kHz]	N [1]	CR [1]	FR [1]
G_max_ [dB]	21	24	23	23
SV @ G_max_ [U]	150	3	30	3
E @ G_max_ [nJ]	12	3	12	12
E_max_ [nJ]	24	E_sat_	35	E_sat_
SV_3dB_ [U]	75–450	1–10	10–150	1.5–5.5
SV_6dB_ [U]	50–600	0.5–12.5	5–300	1–7
E_3dB_ [nJ]	23–3	1.5–24	24–4	5–27
E_6dB_ [nJ]	18–1	0.5–32	12–E_max_–2	2–35
G_sat_ [dB]	n/a	<5	n/a	<1
E_sat_ [nJ]	n/a	60	n/a	53
SV_sat_ [U]	n/a	>30	n/a	>30

## Data Availability

Data sharing is not applicable to this article.
